# High Spinal Block After Intrathecal Mepivacaine in Ankylosing Spondylitis During Total Hip Arthroplasty: A Case Report

**DOI:** 10.7759/cureus.111750

**Published:** 2026-06-29

**Authors:** Juan P Garcia-Mendez, Aamil Patel, Jason Panchamia

**Affiliations:** 1 Department of Anesthesiology and Perioperative Medicine, Mayo Clinic, Rochester, USA

**Keywords:** airway management, anesthetic complications, ankylosing spondylitis, high spinal block, mepivacaine, neuraxial anesthesia, spinal anesthesia, total hip arthroplasty

## Abstract

Ankylosing spondylitis (AS) complicates anesthetic management due to altered spinal anatomy and potential airway difficulties. Neuraxial anesthesia is feasible but technically challenging, with potentially variable drug distribution. High spinal block reports in AS remain limited. This report describes the case of a 58-year-old male patient with AS who underwent elective total hip arthroplasty under spinal anesthesia (3.5 mL 2% isobaric mepivacaine). An initial T11 sensory block rapidly progressed cephalad to C6 within minutes, causing upper extremity weakness, bradycardia, and hypotension. Concern for impending total spinal anesthesia prompted conversion to general anesthesia for airway and hemodynamic control. The patient recovered fully without neurologic deficits. This case illustrates the potential for unpredictable intrathecal anesthetic spread in AS despite standard dosing. Careful monitoring, minimal sedation, and immediate readiness for advanced airway management are crucial during neuraxial anesthesia in patients with AS.

## Introduction

Ankylosing spondylitis (AS) is a chronic inflammatory disease of the axial skeleton with an estimated prevalence of 1% [1]. The progressive ossification of spinal ligaments and joints leads to reduced spinal mobility and, in advanced stages, significant anatomic distortion. While skeletal manifestations are the primary feature, extra-articular involvement may include anterior uveitis, inflammatory bowel disease, pulmonary fibrosis, and, less commonly, neurologic complications such as cauda equina syndrome and dural ectasia [1,2].

These structural changes pose notable anesthetic implications. Neuraxial techniques may be difficult to perform due to restricted spinal mobility and ligamentous calcification [3,4]. Dural ectasia, spinal canal narrowing, and other changes can alter cerebrospinal flow, while airway management can be highly challenging in patients with advanced cervical disease [5]. Consequently, anesthetic planning in this population requires an individualized, cautious approach.

High spinal block is a rare but potentially life-threatening complication of neuraxial anesthesia. It results from the excessive cephalad spread of a local anesthetic above the T4 dermatome, which can lead to cardiovascular and respiratory compromise due to extensive sympathetic and cardiac accelerator nerve fibers blockade, as well as impairment of the respiratory musculature [6]. In the context of AS, existing literature on neuraxial anesthesia has primarily focused on technical difficulties and block failures [4]; reports of high or total spinal phenomena in relation to abnormal intrathecal medication distribution are uncommon. Consequently, the influence of structural abnormalities associated with AS on neuraxial anesthetic spread remains poorly characterized. 

We report a case of an unexpectedly high spinal block following a standard intrathecal dose of mepivacaine in a patient with AS undergoing total hip arthroplasty. This case highlights the potential for abnormal neuraxial blockade progression in patients with atypical spine disease and reinforces the need for vigilant monitoring and readiness to establish airway and cardiovascular interventions. Written informed consent was obtained from the patient for the publication of this case report.

## Case presentation

A 58-year-old male patient with a history of AS presented for an elective left total hip arthroplasty due to advanced osteoarthritis. His AS was diagnosed three years prior, and he was not on active treatment or regular follow-up for the condition. He denied any preexisting neurologic symptoms and had no known extra-articular manifestations. Notably, he had previously undergone a contralateral hip arthroplasty at our institution under spinal anesthesia (using 2.0 mL of 0.5% isobaric bupivacaine) without any complications.

**Figure 1:**
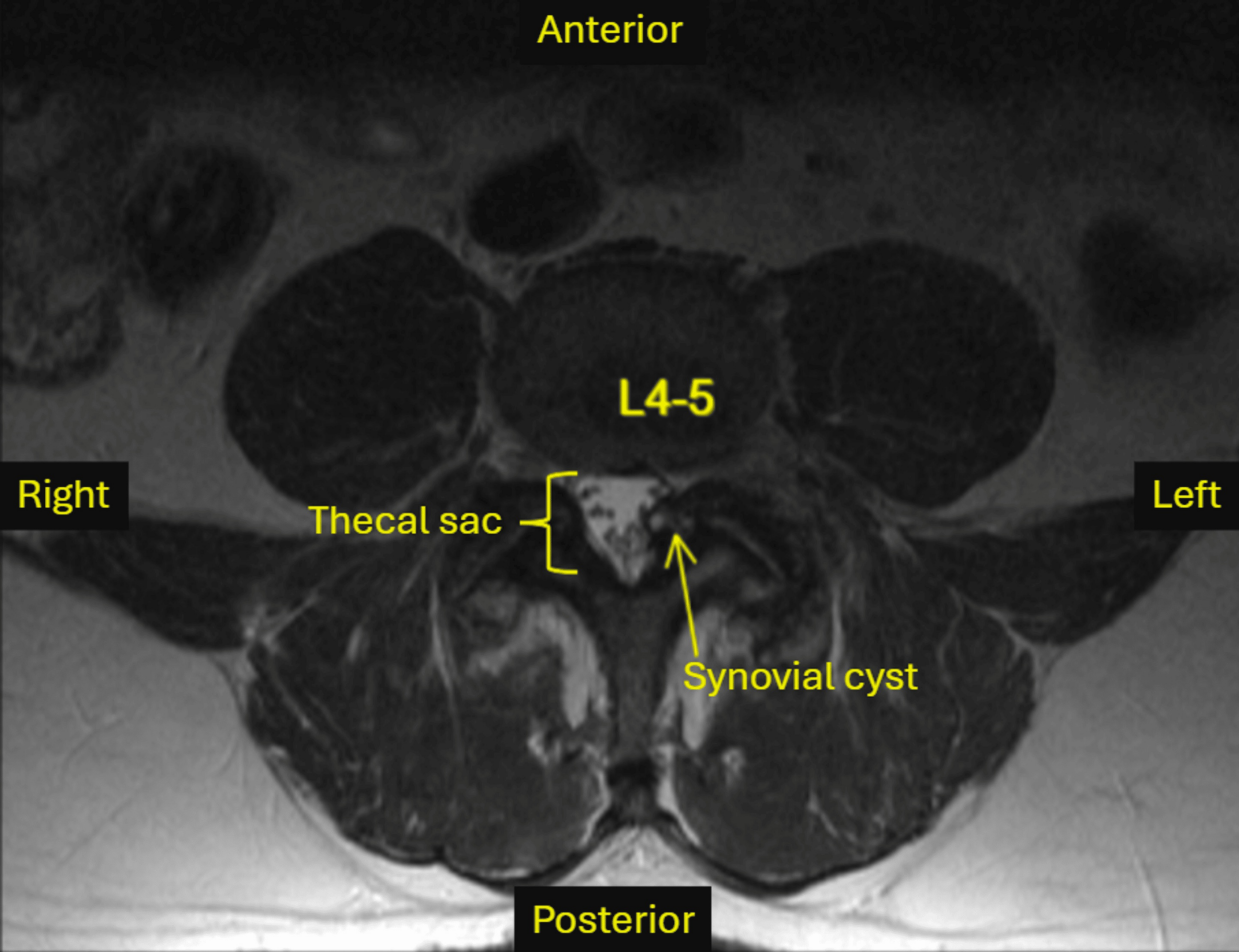
Preoperative lumbar spine MRI Demonstrates the L4-5 left synovial cyst indenting the thecal sac, without causing neural impingement.

Preoperative imaging demonstrated degenerative changes involving the left hip, sacroiliac joints, and lumbar spine, as well as synovial cysts at the L3-4 and L4-5 levels, with the latter indenting the thecal sac without neural impingement (Figure 1).

Following a discussion of anesthetic options, the patient and care team opted for neuraxial anesthesia, guided by patient preference and his history of a successful spinal anesthetic. Given the limited cervical range of motion and no documented airway history, a video laryngoscope and flexible fiberoptic bronchoscope were available in anticipation of a difficult airway, if conversion to general anesthesia was required. Standard American Society of Anesthesiologists monitors, including a pulse oximeter, a noninvasive blood pressure cuff, a continuous electrocardiogram, and capnography, were applied during the procedure.

The spinal anesthetic was performed via a paramedian approach at the L3-L4 interspace using a 22-gauge Whitacre needle. The patient was positioned laterally and received standard monitoring alongside minimal sedation (midazolam 2 mg, fentanyl 50 mcg). Cerebrospinal fluid (CSF) was successfully obtained on the second attempt. Following confirmation of free-flowing CSF, 3.5 mL of isobaric 2% mepivacaine (70 mg) was administered intrathecally.

Three minutes post-injection, the sensory and motor blockade appeared appropriate, with a sensory level assessed at T11. However, over the subsequent five minutes, the block progressed cephalad in a rapid, stepwise fashion to approximately the C6 dermatome as determined by serial assessments using alcohol swabs and blunt needle tips. This progression was characterized by an ascending loss of temperature and pinprick sensation, followed closely by bilateral upper extremity weakness and diminished grip strength. Although the patient remained conscious, his speech progressively slowed, and he appeared lethargic despite receiving no additional sedation. Given the swift cephalad progression and evolving neurologic signs, there was high clinical concern for imminent total spinal anesthesia.

Concurrently, the patient’s heart rate decreased from 58 to 48 beats per minute (bradycardia), and the mean arterial pressure declined from 105 to 60 mmHg (hypotension). He maintained spontaneous ventilation throughout this period and, within the span of 10 minutes, was resuscitated with ephedrine (10 mg followed by a repeat dose of 10 mg), phenylephrine (400 mcg divided into four doses), and glycopyrrolate (0.4 mg). In the setting of hemodynamic instability and the trajectory of the block, the decision was made to convert to general anesthesia to ensure airway protection and strict hemodynamic control. A phenylephrine infusion at a rate of 0.2 mcg/kg/minute was started prior to anesthetic induction. General anesthesia was achieved with a 100 mg bolus of propofol followed by a continuous infusion for maintenance. Endotracheal intubation was achieved without difficulty, and the remainder of the surgical procedure proceeded uneventfully.

Postoperatively, the patient demonstrated a complete resolution of both motor and sensory blockade with no residual neurologic deficits. He was deemed stable and discharged home the same day.

## Discussion

This case describes an unexpectedly high spinal block following the intrathecal administration of mepivacaine in a patient with AS. Intrathecal mepivacaine is frequently utilized for ambulatory lower-extremity procedures due to its rapid onset and highly predictable recovery profile, and prior institutional data support its safety and efficacy for total joint arthroplasty [7]. In the present case, a routine clinical dose resulted in marked cephalad spread, suggesting that anatomical or physiological variables beyond the drug dose itself may have influenced the local anesthetic distribution. Importantly, three years prior, this patient had undergone spinal anesthesia for another orthopedic procedure with 10 mg of isobaric bupivacaine without complication. At that time, 2 mL of bupivacaine was injected, in contrast to 3.5 mL of mepivacaine in our case. Although both represented routine clinical doses for lower-extremity arthroplasty, this increased injectate volume could potentially explain the more abrupt cephalad medication spread in our case. Nonetheless, given the complex array of factors that contribute to intrathecal anesthetic spread, the relative influence of different local anesthetics at equivalent doses cannot be determined.

While the specific challenges of airway instrumentation and neuraxial needle placement in AS are well-documented [3,4,8], the behavior of intrathecal medications within an altered spinal canal is less predictable. High spinal block generally results from factors including injection velocity, baricity, patient positioning, and CSF dynamics [9]. In patients with AS, structural abnormalities such as spinal stenosis, dural ectasia, and localized arachnoiditis- though relatively uncommon - could theoretically alter CSF volume, flow, and drug distribution [8]. In particular, this patient had preoperative radiological evidence of two synovial cysts in the lumbar spine, one of which was indenting the dural sac at the L4-5 level. The impact of lumbar synovial cysts of CSF flow mechanics has not been well characterized. However, their space-occupying nature within the spinal canal could theoretically reduce local CSF volume and contribute to exaggerated cephalad medication spread. Chronic facet joint inflammation and altered spinal biomechanics associated with AS have been proposed as contributors to synovial cyst formation [10,11], although this relationship remains incompletely understood, and its relevance to neuraxial anesthetic spread is unknown. Whether the observed anesthetic spread was related to AS-associated spinal abnormalities, the presence of synovial cysts, or an interaction between multiple anatomic factors cannot be determined from this single case. Furthermore, limited spinal mobility might necessitate non-ideal needle trajectories, which could inadvertently alter the effective level or dynamics of the injection.

Despite these theoretical risks, reports of high spinal phenomena in this population remain scarce, leaving any causal link uncertain. This case illustrates the potential for atypical cephalad spread following routine dosing, though definitive causation cannot be inferred from a single observation.

Determining optimal intrathecal dosing strategies for patients with advanced AS remains challenging. While reducing the anesthetic volume or dose might theoretically limit excessive spread, this approach must be carefully weighed against the risk of providing inadequate surgical anesthesia, particularly given that drug distribution is multifactorial and not solely volume dependent. Peripheral nerve blocks may serve as a viable alternative strategy in selected cases where neuraxial techniques are deemed too unpredictable [12].

Practically, this case reinforces several clinical safeguards. Utilizing minimal preoperative sedation during neuraxial anesthesia allows the clinician to continuously monitor the patient's neurologic baseline, facilitating the early recognition of an ascending block. Additionally, maintaining an immediate readiness for advanced airway management is paramount, as early recognition of the progressing block in this instance allowed for a controlled, timely conversion to general anesthesia before respiratory failure occurred.

## Conclusions

This case demonstrates the potential for unpredictable variability in intrathecal local anesthetic spread in patients with AS, even when utilizing standard dosing and techniques. No specific recommendations exist regarding local anesthetic dose reduction in patients with AS, and the decision to do so must consider the risk of inadequate surgical anesthesia. Although the causal link between AS and high spinal block remains uncertain, the present case highlights several considerations, including the value of closely monitoring spinal block level, maintaining a high index of suspicion for atypical block progression, and preparing for airway and hemodynamic interventions, as well as timely conversion to general anesthesia should a high spinal block occur. Additional reports are needed to further characterize the relationship between AS and abnormal CSF flow patterns with an increased risk of local anesthetic cephalad spread.
